# Annealing induced atomic rearrangements on (Ga,In) (N,As) probed by hard X-ray photoelectron spectroscopy and X-ray absorption fine structure

**DOI:** 10.1038/s41598-018-23941-y

**Published:** 2018-04-13

**Authors:** Fumitaro Ishikawa, Kotaro Higashi, Satoshi Fuyuno, Masato Morifuji, Masahiko Kondow, Achim Trampert

**Affiliations:** 1 Graduate School of Engineering, Osaka University, 2-1 Yamadaoka, Suita, Osaka 565-0871 Japan; 2 Graduate School of Science and Engineering, Ehime University, 3 Bunkyo-cho, Matsuyama, Ehime 790-8577 Japan; 3 Paul-Drude-Institute für Festköperelektronik, Hausvogteiplatz 5-7, 10117 Berlin, Germany; 4 Present Address: Innovation Research Center for Fuel Cells, The University of Electro-Communications, 1-5-1 Chofugaoka, Chofu, Tokyo 182-8285 Japan

## Abstract

We study the effects of annealing on (Ga_0.64_,In_0.36_) (N_0.045_,As_0.955_) using hard X-ray photoelectron spectroscopy and X-ray absorption fine structure measurements. We observed surface oxidation and termination of the N-As bond defects caused by the annealing process. Specifically, we observed a characteristic chemical shift towards lower binding energies in the photoelectron spectra related to In. This phenomenon appears to be caused by the atomic arrangement, which produces increased In-N bond configurations within the matrix, as indicated by the X-ray absorption fine structure measurements. The reduction in the binding energies of group-III In, which occurs concomitantly with the atomic rearrangements of the matrix, causes the differences in the electronic properties of the system before and after annealing.

## Introduction

Dilute nitride semiconductors are interesting material systems because of the tunability of their band gaps and strain conditions, and they are thus expected to be used in next generation optical devices that operate at infrared wavelengths that have previously been unachievable^1–4^. For example, these nitrides have recently been recognized as suitable materials for use in high-efficiency solar cells because their absorption edges overlap with the infrared region of the solar spectrum^5–7^. However, it is well known that as-grown samples of these dilute nitrides contain considerable numbers of defects within the crystals, including interstitials^8,9^, antisite defects^10^, and vacancies^11^, which occur because of the relatively low temperature conditions required for dilute nitride film growth. Reduction of these defects is essential to provide improved optical and electrical properties for these materials. Therefore, post-growth annealing is a vital process for device applications of these materials^9^. However, annealing can induce an unexpected and strong blueshift in the emitting wavelength in these material systems, which is undesirable for applications in the infrared regime. The origin of this blueshift was previously indicated to be due to atomic rearrangement of the constituent elements around N investigated by synchrotron x-ray measurements of X-ray absorption near edge structure (XANES) and X-ray absorption fine structure (XAFS)^12,13^. Optical investigations of photoluminescence, photoreflectance, and electroreflectance spectroscopies quantitatively evaluated the distribution of N-In nearest-neighbor states^13–17^. Those reports suggests the driving force of the thermodynamic equilibration of the N-In nearest-neighbor bonding in the material toward highly In-coordinated states, from an as-grown material having a nearly random bonding arrangement dominated by N-Ga bonds^13–17^. The unique properties of these defects can also be applied to form advanced functional devices, such as a spin-filter that operates at room temperature^8^. The characterization of these defects and control of the number of defects produced by annealing, have been studied over the past few decades^9,12,13,18^. X-ray photoemission spectroscopy is widely used to investigate the electronic states of matter, including the aforementioned defects in dilute nitride systems^19,20^. In conventional X-ray photoemission spectroscopy using Al or Mg anodes and X-ray sources with energies of 1–1.5 keV, the escape depth of the photoelectrons is generally several nanometers. Therefore, the signals in conventional X-ray photoemission spectroscopy are strongly affected by both the surface conditions and the out-diffusion of the elements, particularly in the post-annealing case^20^. In addition, the high photon fluxes realized by undulators in third-generation synchrotron light sources and the development of the electron energy analyzer for high-kinetic-energy electrons have recently made practical high-resolution hard X-ray photoelectron spectroscopy (HAXPES) possible. The increased escape depths of photoelectrons, which range to depths of several tens of nm, with higher kinetic energies will enable nondestructive studies of bulk materials, nanoscale buried layers and their interfaces^21,22^. XAFS techniques can provide nondestructive analysis of the electronic and local structures of specific atoms. Here, we use a depth-resolved XAFS technique to analyze the effects of annealing on the sample^23^. This technique has been used to clarify the atomic structure without substrate information and provides detailed microstructural information about the buried epitaxial layers contained within the sample. These developed HAXPES and XAFS investigations can also compliment the preceding reports from Lordi *et al*.’s^12,13^, through the direct observation of chemical bonding in (Ga,In) (N,As) and the nearest-neighbor configurations around Ga and As due to its depth-resolved material selectivity and greater signal sensitivities. In this report, we analyze the effects of annealing on the microscopic crystal characteristics of (Ga,In) (N,As)/Ga(N,As) material systems by comparison of the electronic states and bond configurations that were obtained by HAXPES and XAFS.

## HAXPES Results

**Figure 1:**
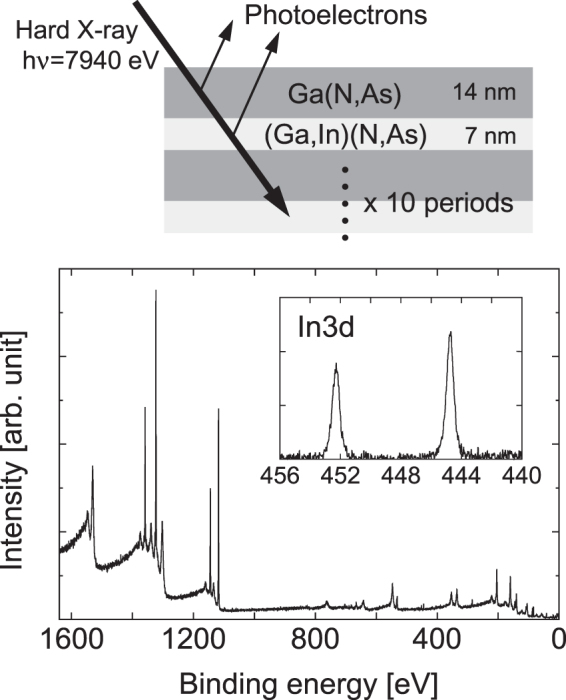
Wide range HAXPES spectra. The conditions close to the surface are illustrated schematically. The inset shows the spectrum close to the In 3*d* region.

In this report, we study a sample consisted of ten periods of [(Ga,In) (N,As) (8 nm)/Ga(N,As) (14 nm)] with constituent compositions of 36% In and 4.5% N for the (Ga,In) (N,As) wells and 0.8% N for the Ga(N,As) barriers, as shown in Fig. 1. To investigate the effects of annealing on the material, we made comparison for the sample before (as-grown) and after rapid thermal annealing (RTA), which was performed for 1 min under a N_2_ atmosphere at 730 °C. In the HAXPES spectra in this report, when a clear fit has been obtained, the experimental data points are indicated by dots and the fitting curves are superimposed on those dots as colored lines, unless otherwise stated. Figure 1 shows the wide range HAXPES spectrum of the as-grown sample. The conditions close to the surface are also illustrated schematically. As shown in the inset in the figure, distinct In-related peaks are observed. These peaks indicate that the probing depth of this measurement is sufficient for analysis of the buried (Ga,In) (N,As) layer below the 14 nm-thick Ga(N,As) surface. In this section, we present representative HAXPES spectra to enable discussion of the findings of this study. The other HAXPES spectra, including those for the light elements of C, N, and O, along with the spectra around the Ga and In orbitals, are summarized in the Appendix.

**Figure 2:**
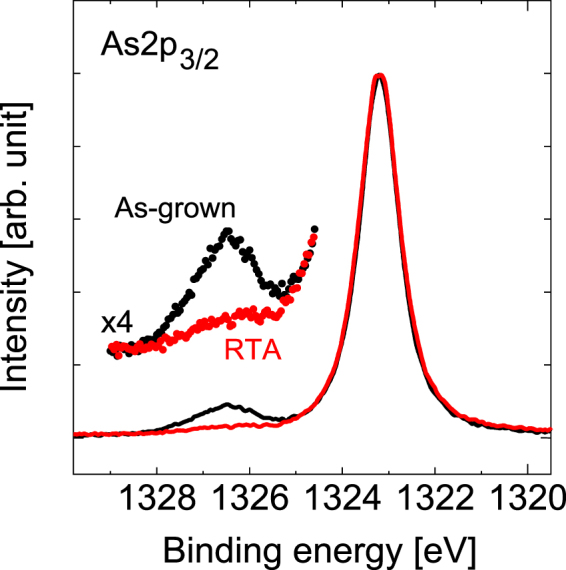
HAXPES spectra of As 2*p*_3/2_ spectral regions before and after RTA.

**Figure 3:**
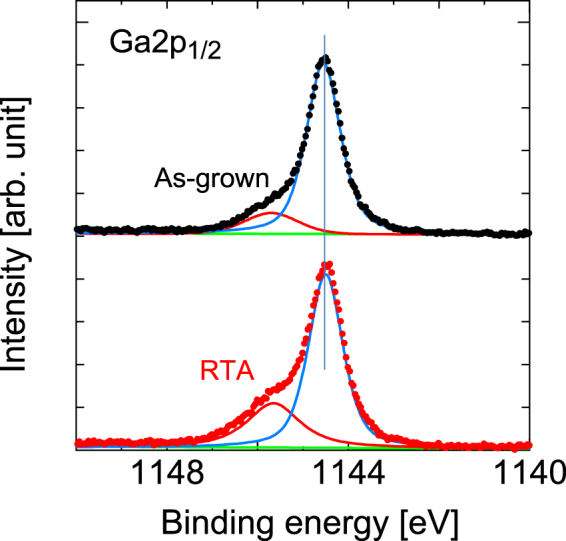
HAXPES spectra of Ga 2*p*_1/2_ regions before and after RTA. The vertical line serves as a guide for the eye to aid in examination of the peak shift.

Figure 2 shows the HAXPES spectra at the As 2*p*_3/2_ regions of the samples before and after RTA. The peaks at 1323.2 eV and 1326.55 eV can be assigned to Ga-As^24^ and either As-O^24^ or N-As^25,26^, respectively. Figure 3 shows the HAXPES spectra at the Ga 2*p*_1/2_ regions of the samples before and after RTA. The main peak at 1144.6 eV appears to originate from the Ga-As bond^24,27–29^. The sub-peak observed at 1145.8 eV can then be assigned to the Ga-O bond. As shown in Fig. 2, the main Ga-As peak is observed at 1323.2 eV, while a small sub-peak occurs around 1326.6 eV under as-grown conditions. The sub-peak may stem from the As-O or N-As configurations. However, this sub-peak vanished after RTA. Additionally, the intensity of the peak that was assigned to Ga-O bonding increased after RTA, as shown in Fig. 3.

These results indicate the presence of increased surface oxides after RTA, and these oxides are unlikely to decrease. Consequently, the sub-peak shown in Fig. 2 is related to the N-As configurations^30^. The group V-group V bond is a defect in itself. The as-grown sample would have N-As bonds with a concentration that is close to the detection limit. RTA diminishes the numbers of these defects, which causes their concentration to fall below the detection limit.

**Figure 4:**
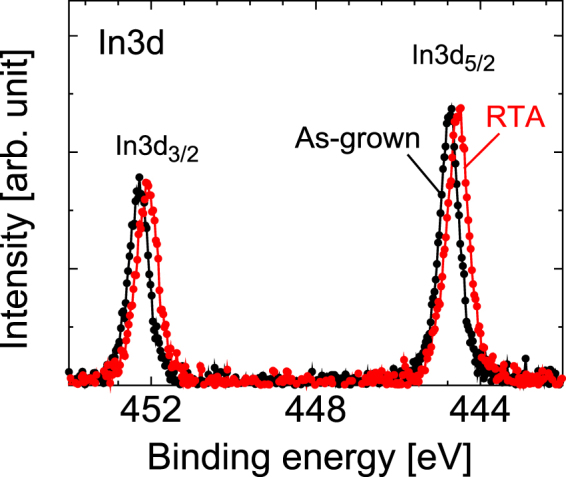
HAXPES spectra of In 3*d*_3/2_ and In 3*d*_5/2_ regions before and after RTA. The lines are used to connect the dots of the experimental data points.

Figure 4 shows the HAXPES spectra measured at the In 3*d* regions before and after RTA. The peaks shown at 452.3 eV and 444.8 eV can be assigned to In 3*d*_3/2_ and In 3*d*_5/2_, respectively, and are related to the In-As bond^31^. Specifically, the peak positions of both peaks in the figure are clearly different before and after RTA. This suggests that there is a characteristic change in the electronic states around In. Further discussions involving comparison of the overall HAXPES and subsequent XAFS results will be provided later in the paper.

## XAFS Results

**Figure 5:**
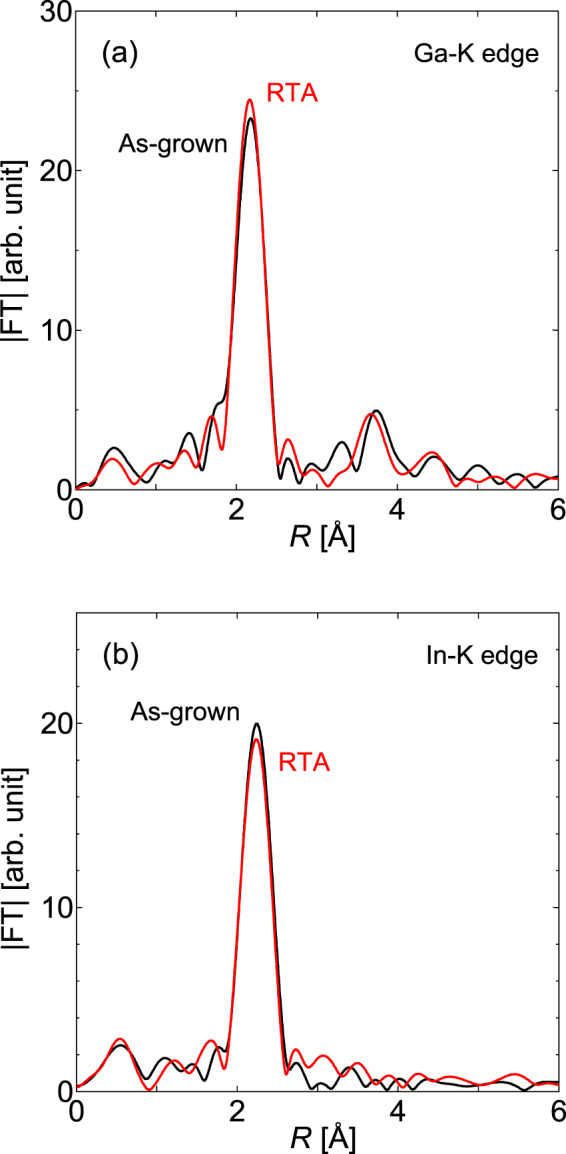
RSFs around the (**a**) Ga K-edge and (**b**) In K-edge before and after RTA that were obtained via depth-resolved and standard fluorescent XAFS measurements, respectively. The X-ray polarization was along the [001] direction during the measurements.

Figure 5(a,b) show radial structure functions (RSFs) around the Ga K- and In K-edges, respectively, that were obtained via XAFS measurements. Note that In is only present in the 10-period (Ga,In) (N,As) quantum well layers, and the Ga information was extracted from approximately 40 nm of the top-most surface using depth-resolved measurements, as described in the Supplemental Material. In both RSFs, we observe a sharp peak at approximately 2.1 Å, which corresponds to the first-nearest neighbor As bond shell. Additionally, the intensity of the main peak increases for Ga but the corresponding intensity decreases for In after RTA. When we focus on the atomic configurations around In, the results above indicate that RTA induces the reduction in the number of In-As bonds and thus also induces the corresponding increase in the number of In-N bonds.

**Figure 6:**
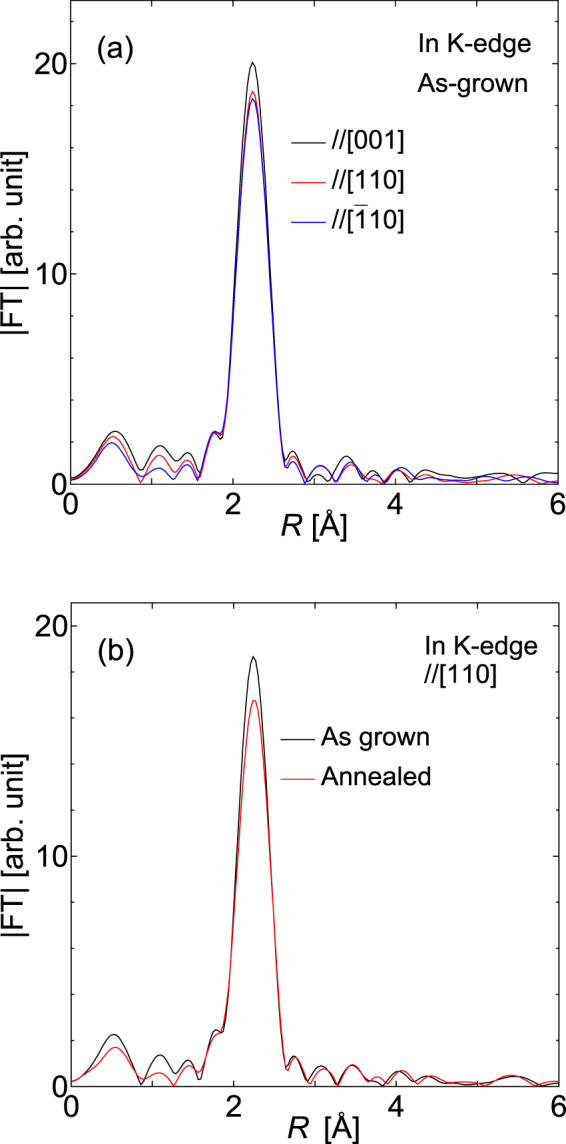
(**a**) RSFs around the In K-edge for the different incident X-ray polarizations used for the measurements. (**b**) In K-edge RSFs before and after RTA for incident X-ray polarization along the [110] direction.

Figure 6(a) shows the polarization direction dependence of the In K-edge RSF of the sample. We have measured the spectral variations with the incident X-ray polarization directions as indicated in the figure, which shows the difference between the in-plane direction (corresponding to the incident X-ray polarizations of //[110] and [−110]) and the out-of-plane growth direction (//[001]). In the figure, the peak intensity differs between the in-plane directions and the out-of-plane [001] direction because of the atomic structure. Figure 6(b) shows the RSFs before and after RTA for incident X-rays polarized in the [110] direction. The RTA induced a reduction in the In-As peak intensity. The phenomenon is more pronounced here than in the case that was observed in Fig. 5(b).

**Figure 7:**
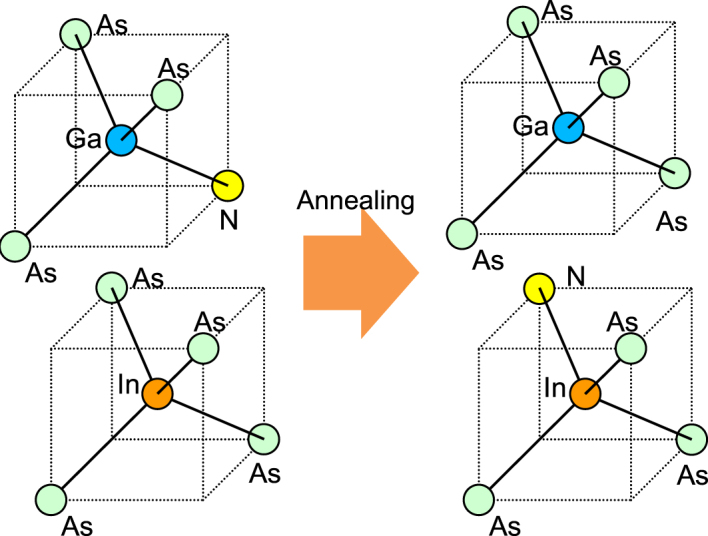
Schematic illustrations of atomic configurations considered around Ga and In with adjacent N before and after RTA.

The results shown in Figs 5 and 6 indicate the increase in the Ga-As bonding configuration and the decrease in the In-As bonding configuration that occur after RTA. Because we can obtain genuine information from the (Ga,In) (N,As) layer from the In K-edge spectra without it being affected by the Ga(N,As) barrier, we focus first on the reduction in the number of In-As configurations. The results for the Ga K-edge complement and support the findings above. The greater reduction in the In-As peak intensity that was observed in the in-plane [110] direction in Fig. 6(b) when compared with the case for the [001] direction shown in Fig. 5(b) indicates the presence of anisotropic annealing-induced atomic rearrangements. The fitting results for the XAFS measurements, which are summarized in Figs S3–S7 and Tables SI and SII in the Supplemental Materials, support the above conclusions. The coordination number of the In atoms apparently decreases from 3.8 to the 3.5–3.6 range after RTA. In contrast, the coordination number of Ga is either maintained or increases slightly around the initial value of 3.9 after RTA. Based on the results above, the atomic configuration considered here, including the N atoms before and after RTA, is illustrated schematically in Fig. 7. As shown in the figure, the bond configurations of the N atoms that are adjacent to In increased after annealing while the corresponding bond configurations adjacent to Ga were reduced. This phenomenon can be expressed using the atomic rearrangements that were proposed by Lordi *et al*.^12,13^, which will be discussed further in the next section.

## Discussions

### Elimination of N-As bond defects and surface oxidation

**Figure 8:**
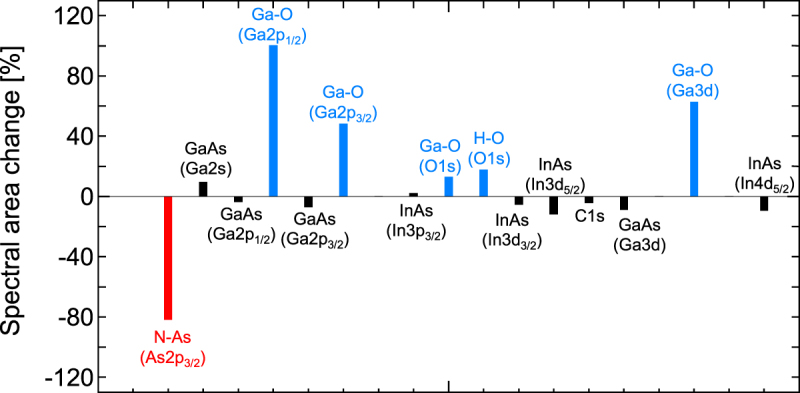
Spectral area changes with respect to the representative bonding configurations before and after RTA. The values are extracted from the comparison of integrated intensities for the respective bonding configuration peaks before and after RTA shown in Figs 3, 4 and 11–15.

We summarize the spectral peak area changes in the characteristic bond configurations that were induced by RTA in Fig. 8. As shown in the figure, the bond configurations related to the constituents, i.e., the group III to group V bonds, did not show any significant differences before and after RTA. In contrast, the N-As bond configuration that was observed in the As 2*p*_3/2_ spectrum showed a significant reduction in intensity after RTA. In addition, the oxide related spectra from the Ga-O and H-O bonding configurations observed in the O1*s* spectrum showed remarkable increases in all their peak areas. The N-As bond is in itself a defect and is most likely to originate from a split interstitial consisting of a nitrogen atom and an arsenic atom on a single arsenic lattice site^30,32,33^. These split interstitial defects have been predicted theoretically as defects with energetically favorable configurations^32,33^, and these predictions have been supported experimentally^34–37^. Consequently, RTA appears to terminate these defects while concomitantly causing surface oxidation to progress^30^.

### In core level shifts with atomic rearrangement

**Figure 9:**
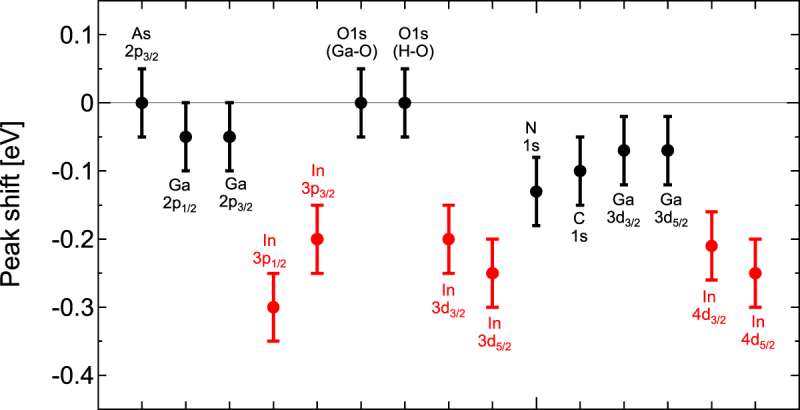
Peak shifts with respect to the core level binding energies before and after RTA. The values are extracted from the comparison of peak positions for the respective bonding configuration peaks before and after RTA shown in Figs 3, 4 and 11–15 (In Appendix).

**Figure 10:**
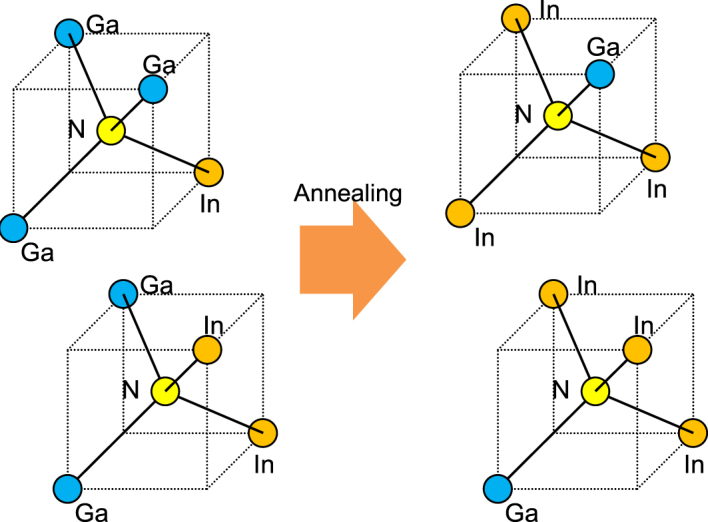
Schematic illustration of atomic configurations considered around N before and after RTA.

Figure 9 compiles the HAXPES spectral peak shifts that were induced by RTA. We only observe significant spectral peak shifts for the In-related peaks, indicating a reduction in the binding energy. The RTA thus has a peculiar effect on the electronic states of In. To consider the origins of this phenomenon, we first discuss the atomic configurations around In based on the XAFS results. The results shown in Figs 5 and 7 indicate that the bond configuration of In around N is changed by the annealing process. This phenomenon can be expressed in terms of the atomic rearrangements^12,13,38^. In the XAFS results, we observed possible atomic rearrangements around the majority group-V As atoms and that should have an effect on the N atoms. This indicates that there is an increase in the large number of In-N bonds for the minority group-V constituent N atoms after the annealing step. Our findings follow the results of Lordi *et al*.^12,13^. They found that the as-grown material consisted of near-random atomic distributions, whereas annealed samples showed segregation of the In atoms towards N^12,13^. These thermodynamically stable configurations minimize the local strain in the matrix, inducing atomic rearrangement in addition to the random distribution^12,13,38^. This is also accepted to be the origin of the blueshift in the wavelength emitted from the material. When we consider the 36% In concentration in the (Ga,In) (N,As) layer, the numbers of the configurations with one or two In atoms around the N atom would initially be roughly similar under random distribution conditions. However, the increase in the number of In-N bonds suggests that, when the most energetically favorable configurations suggested by Lordi *et al*.^12,13^ are taken into account, the configuration with three In atoms around N could have the largest numbers within the matrix after RTA. The atomic configurations considered around N before and after RTA are illustrated schematically in Fig. 10.

As described above, RTA strongly affects the electronic status of In and this results in a chemical shift of the In peak towards the lower binding energy side in HXPES. This shift can be explained by the increase in electron charge density of the In atoms that is caused by the reduction in the interactions between In and the surrounding atoms^39^. Because the conduction band edge basically originates from the *s*-orbitals of the group-III elements in the III-V semiconductor, the reduced interactions between the group-III In atoms and the surrounding atoms may induce a reduction in the group-V N-induced band gap modifications^40^. The modified electronic states of the group-III In can thus explain the blueshift that occurs in this material system after annealing.

## Summary

We studied the effects of annealing on (Ga_0.64_,In_0.36_) (N_0.045_,As_0.955_) using HAXPES and XAFS. We observed surface oxidation with concomitant progress in termination of the N-As bond defects as a result of the annealing step. The characteristic chemical shift was observed solely for the photoelectron spectra related to In at energies ranging towards the lower binding energies between the adjacent atoms, and this was observed most specifically in the HAXPES spectra. The XAFS results indicate that the reduction in the binding energy around In is likely to be caused by the atomic rearrangement, which leads to increased In-N bond configurations within the matrix. The reduction of the group-III In binding energies may explain the blueshift in the emission wavelength of this material system.

## Methods

We investigated (Ga,In) (N,As)/Ga(N,As) sample that was grown on semi-insulating GaAs(001) substrates by molecular beam epitaxy^41^. Conventional solid-source effusion cells were used for Ga and In, and an As-valved cracker cell was operated in the As_4_ mode. Nitrogen was supplied by an radio frequency plasma source. The substrate temperature during growth was monitored with a thermocouple, which has been calibrated by combining the GaAs oxide desorption temperature of 580 °C, the In melting point of 160 °C, and the transition points of reflection high-energy electron diffraction (RHEED) patterns^42^. Prior to the growth of the (Ga,In) (N,As)/Ga(N,As) MQW, a 300 nm thick undoped GaAs buffer layer was grown at 560 °C. For the growth of the MQW, the fluxes of all the elements were fixed throughout this study. A low As BEP of 8 × 10^−7^ Torr was used, which corresponds to a V/III BEP ratio of 5. The atomic V/III flux ratio was estimated to be 3. The growth rates were 0.24 *μ*m/h for the Ga(N,As) barriers, and 0.4 *μ*m/h for the (Ga,In) (N,As) quantum wells (QWs). The In content was kept at 36%. The N concentration was kept slightly higher than 4% by assuming an identical incorporation for the ternary and quaternary material. The Ga(N,As) barriers were grown by closing the shutters of both the In and the N cells in order to obtain a large band offset. However, a small amount of about 0.6–0.8% N was incorporated into the GaAs layers in our case, due to the residual amount of atomic N diffusing into the growth chamber around the closed shutter. The sample consisted of ten periods of [(Ga,In) (N,As) (8 nm)/Ga(N,As) (14 nm)] with constituent compositions of 36% In and 4.5% N for the (Ga,In) (N,As) wells and 0.8% N for the Ga(N,As) barriers, as shown in Fig. 1. Note that the In and N concentrations are among the largest concentrations of these constituents within the (Ga,In) (N,As) materials to be reported to date^41,43^. To investigate the effects of annealing on the materials, RTA was performed for 1 min under a N_2_ atmosphere at 730 °C employing a halogen lamp heated rapid thermal processing system (JetFirst, SEMCO, France) after growth; these conditions appropriately enhance the luminescence efficiency of the samples^41^. During RTA, the samples were proximity-capped using a GaAs wafer to prevent evaporation of the constituent elements.

All measurements described below were performed at room temperature. The HAXPES measurements were carried out at the BL46XU beamline of SPring-8^30^. The undulator X-rays were monochromatized using a Si (111) double-crystal monochromator and were further monochromatized using a Si (111) channel-cut post-monochromator. The photon energy was set at 7.94 keV using the Si (444) reflections of the post-monochromator. The photoelectron energy analysis was performed using the VG-Scienta R-4000, and the pass energy was set to 200 eV. The X-ray angle of incidence and the photoelectron take-off-angle with respect to the sample surface were 10° and 80°, respectively. The binding energy was calibrated using the 4*f* core level energy that was observed from a directly deposited Au thin film on the sample surface. For qualitative analysis, the observed peak intensities were normalized with respect to the peak intensity of the dominant group-V element: the As 2*p*_3/2_ peak. Deconvolution of the peaks that were superposed in the spectra was performed by curve fitting using a sum function of Gaussian and Lorentzian profiles with a Gaussian percentage of approximately 80%. No special surface cleaning processes were carried out before the HAXPES measurements to prevent sample damage and to obtain information about the bare sample characteristics.

The XAFS experiments were performed on the BL37XU beamline of SPring-8^23,44,45^. The X-ray beam that was monochromatized by the Si (111) double-crystal was used to irradiate the sample surface after passing through a slit with dimensions of 0.5 mm x 0.5 mm. The angle of incidence of the X-ray on the sample was controlled by considering the polarization of the X-ray beam. A two-dimensional pixel array detector (Pilatus 100 K), which was positioned parallel to the sample plane, was used to detect the emitted fluorescence. The distance between the irradiation point on the sample surface and the detector window was 172 mm. The detection angle resolution of a one-pixel array with a width of 172 *μ*m is 1 mrad.

The fluorescence intensity that is detected at each pixel includes the different weights of contributions from the layers located at different depths that are dependent on the detection angle. Further information about the XAFS apparatus and its measurement modes is given in the Supplemental Materials. To measure the Ga K-edge, we used depth-resolved XAFS measurements; otherwise, the signal would also contain the fluorescence from the GaAs substrate. To investigate In, we performed standard fluorescence mode XAFS measurements. Because In is only included in the (Ga,In) (N,As) layer, genuine information can thus be obtained from the (Ga,In) (N,As) layer.

The extraction of the extended X-ray absorption fine structure (EXAFS) oscillation from the spectra, normalization via an edge jump, Fourier transformation, and curve-fitting analysis were carried out using the REX2000 analytical package via a standard procedure^46^. The EXAFS oscillation was analyzed using the following EXAFS oscillation function *χ*(*k*): $\chi (k)=Nf(k)\,\exp \,(\,-\,2{\sigma }^{2}{k}^{2}-2r/\lambda )\,\sin \,\mathrm{(2}kr+\varphi (k))/k{r}^{2}$, where the electron wave vector *k* is defined as *k* = [2*m*(*E* − *E*_0_)/*ħ*]^1/2^. *N* and *r* are the number of atoms and the distance between the absorbing atom and the corresponding shell, respectively; *f*(*k*) is the backscattering amplitude, *σ* is the Debye-Waller factor, *λ* is the mean free path of the photoelectrons, *ϕ* is the phase shift, *m* is the electron mass, and *E*_0_ is the energy at the absorption edge. *f*(*k*) and *ϕ*(*k*) were calculated using the McKale database^46^. We then obtained the parameters *N*, *E*_0_, *r*, *σ*, and *λ* by curve fitting. To obtain the R-factor (a measure of the goodness-of-fit), we used ${\rm{R}}=\sqrt{{\rm{\Sigma }}\,{({\chi }_{\exp }-{\chi }_{{\rm{fit}}})}^{2}}/\sqrt{{\rm{\Sigma }}\,{({\chi }_{\exp })}^{2}}$, where *χ*_exp_ and *χ*_fit_ are the experimentally and theoretically obtained values of *χ*, respectively.

## Appendix A: HAXPES spectra

**Figure 11:**
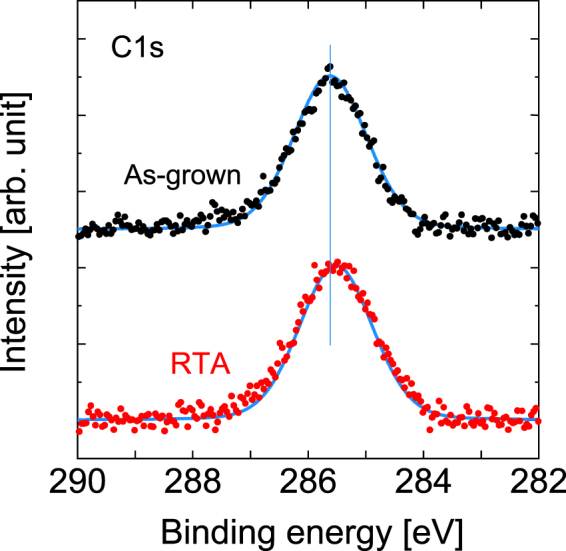
HAXPES spectra of C 1*s* spectral regions before and after RTA. The vertical line acts as a guide for the eye to aid in examination of the peak shift.

Figure 11 shows the HAXPES spectra in the C 1*s* regions. We can see that a single peak originates from C 1*s* at 285.7 eV^47^. We do not see any distinct differences between the features of these spectra before and after annealing.

**Figure 12:**
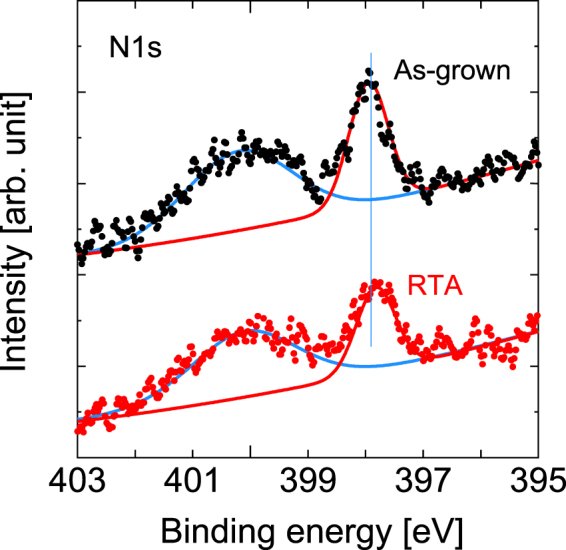
HAXPES spectra of the N 1*s* spectral regions before and after RTA. The vertical line acts a guide for the eye to aid in examination of the peak shift.

Figure 12 shows the HAXPES spectra in the N 1*s* regions. The peak at 397.8 eV originates from N 1*s*, which is mainly bonded to Ga^19,48,49^. The other peak at 400.1 eV is likely to be related to Ga-LMM Auger electrons^49^. Note that the HAXPES measurements are not sensitive to lighter elements. Therefore, we show this N 1*s* solely for the purposes of qualitative discussions or reference. We omitted the results related to this spectrum from the quantitative discussions contained in the main text.

**Figure 13:**
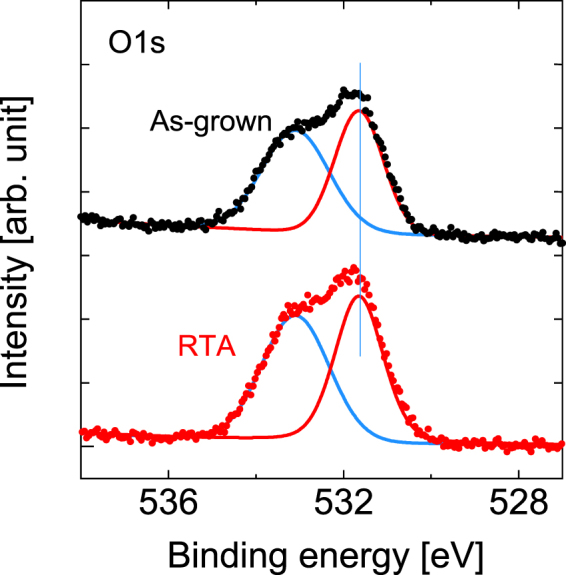
HAXPES spectra of the O 1*s* spectral regions before and after RTA. The vertical line acts a guide for the eye to aid in examination of the peak shift.

In Fig. 13, the peak at 533.1 eV originates from the atmospheric H-O bond that is commonly observed at the surface of matter^24^. The other peak at 531.7 eV results from the superposition of the oxides of the constituent elements, i.e., where the dominant component, which comes from the Ga-O configuration^50,51^, is mixed with the minor components from the In-O^52^ and As-O^53^ configurations.

**Figure 14:**
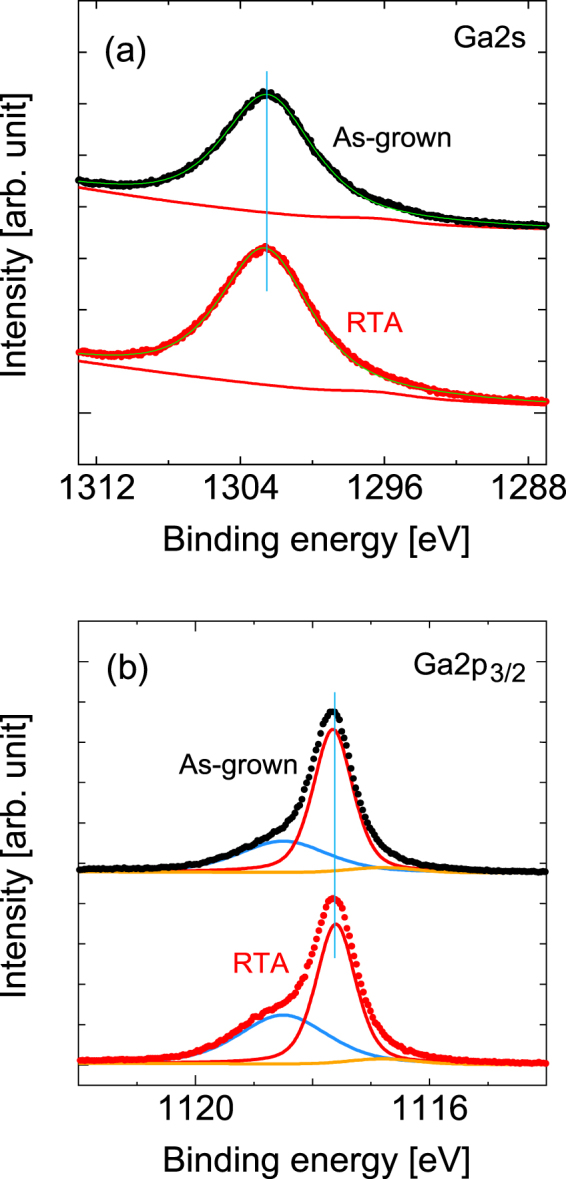
HAXPES spectra of (**a**) Ga 2*s* and (**b**) Ga 2*p*_3/2_ spectral regions. The vertical lines act as guides for the eye to aid in examination of the peak shift.

Figure 14(a) shows the HAXPES spectra in the Ga 2*s* regions of the sample before and after RTA. The main peak at 1302.5 eV originates from the Ga-As bond^24^. We also observed a weak peak at 1296.5 eV that induces an overall spectral line shape that is tailored to the lower energy side, although the origin of this peak is uncertain. Figure 14(b) shows the HAXPES spectra in the Ga 2*p*_3/2_ regions. The weak signal at 1116.8 eV was attributed to Ga^27^. The distinctive peaks that occur at 1117.65 eV and 1118.5 eV can be assigned to Ga-As^28^ and Ga-O bonds^27^, respectively. As shown in Fig. 3, we found an increase in the peak intensity on the oxide related peak.

**Figure 15:**
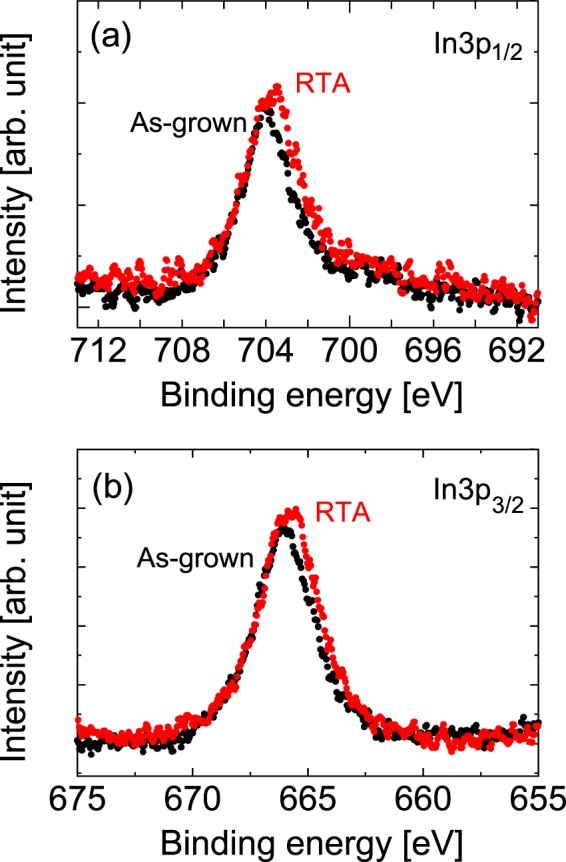
HAXPES spectra of (**a**) In 3*p*_1/2_ and (**b**) In 3*p*_3/2_ spectral regions measured before and after RTA.

Figure 15(a,b) show the HAXPES spectra at the In 3*p*_1/2_ and In 3*p*_3/2_ regions, respectively, as measured before and after RTA. These peaks can be assigned to the In-As bond^31^. Note here that the signal-to-noise ratio is rather worse than that which originated from peaks ranging from As to Ga because of the small In content when compared with the other atoms. However, we can clearly see the peak shift before and after RTA that was also seen in the In-related peak for In 3*p*_1/2_ in Fig. 4.

**Figure 16:**
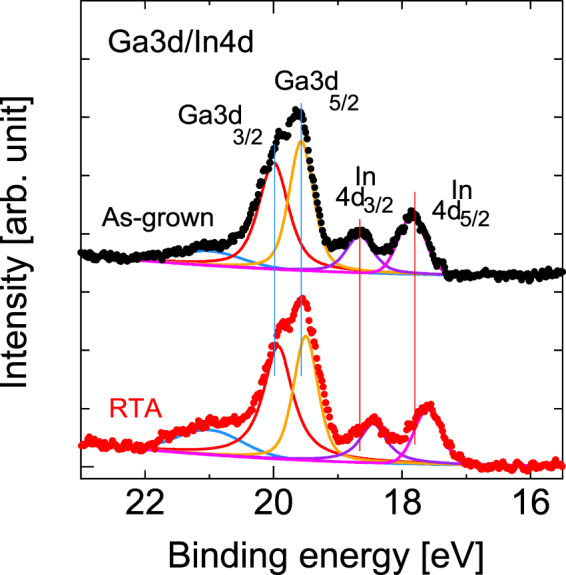
HAXPES spectra of the Ga 3*d* and In 4*d* spectral regions measured before and after RTA. The vertical lines act as guides for the eye to aid in examination of the peak shift.

Figure 16 shows the HAXPES spectra measured at the Ga 3*d*-In 4*d* regions before and after RTA. The peaks at 17.8 eV and 18.7 eV can be assigned to In-As (In 4*d*_5/2_) and In-As (In 4*d*_3/2_), respectively^31^. Two components that were assigned to the Ga 3*d*_5/2_ and Ga 3*d*_3/2_ peaks in the Ga-As bonding configuration are noticeable at 19.6 eV and 20.0 eV, respectively, because of the high energy resolution of the HAXPES measurement system that was used^50,54,55^. The peak shown at 21.0 eV is attributed to Ga-O^50,54,55^. As discussed earlier, we observed enhancement of the Ga-O related peaks, as shown in Figs 3 and 14. Most specifically, the two In-related peaks shifted obviously after RTA by more than 0.2 eV towards the lower energy side. In contrast, the shifts in the other peaks were related to the Ga marginal.

## Electronic supplementary material

Supplementary information

## References

[1] Ed. Henini M (2005). Dilute Nitride Semiconductors.

[2] Ed. Buyanova IA, Chen WM (2004). Physics and Applications of Dilute Nitrides.

[3] Tansu N, Yeh JY, Mawst LJ (2004). Physics and characteristics of high performance 1200 nm InGaAs and 1300–1400 nm InGaAsN quantum well lasers obtained by metal-organic chemical vapour deposition. J. Phys.: Condens. Matter.

[4] Mawst LJ (2008). MOCVD-grown dilute nitride type II quantum wells. IEEE J. Sel. Topics Quantum Electron..

[5] Langer, F., Perl, S., Höfling, S. & Kamp, M. Graded band gap GaInNAs solar cells. *Appl*. *Phys*. *Lett*. **106**, 233902-1-6 (2015).

[6] Green MA, Emery K, Hishikawa Y, Warta W, Dunlop ED (2015). Solar cell efficiency tables (Version 45). Prog. Photovolt. Res. Appl..

[7] Wiemer M, Sabnis V, Yuen H (2011). 43.5% efficient lattice matched solar cells. Proc. SPIE.

[8] Wang XJ (2009). Room-temperature defect-engineered spin filter based on a non-magnetic semiconductor. Nature Mater..

[9] Spruytte SG (2001). Incorporation of nitrogen in nitride-arsenides: Origin of improved luminescence efficiency after anneal. J. Appl. Phys..

[10] Thinh NQ, Buyanova IA, Chen WM, Xin HP, Tu CW (2001). Formation of nonradiative defects in molecular beam epitaxial GaN_*x*_As_1−*x*_ studied by optically detected magnetic resonance. Appl. Phys. Lett..

[11] Toivonen J (2003). Observation of defect complexes containing Ga vacancies in GaAsN. Appl. Phys. Lett..

[12] Lordi, V. *et al*. Nearest-neighbor configuration in (GaIn) (NAs) probed by X-ray absorption spectroscopy. *Phys*. *Rev*. *Lett*. **90**, 145505-1-4 (2003). 10.1103/PhysRevLett.90.145505 12731929

[13] Lordi, V *et al*. Nearest-neighbor distributions in Ga_1−*x*_In_*x*_N_*y*_As_1−*y*_ and Ga_1−*x*_In_*x*_N_*y*_As_1−*y*−*z*_Sb_*z*_ thin films upon annealing. *Phys*. *Rev*. *B* **72**, 125309-1-8 (2005).

[14] Klar, P. J. *et al*. (Ga, In) (N, As)-fine structure of the band gap due to nearest-neighbor configurations of the isovalent nitrogen. *Phys*. *Rev*. *B* **64**, 121203(R)-1-4 (2001).

[15] Kudrawiec R, Sȩk G, Misiewicz J, Gollub D, Forchel A (2003). Explanation of annealing-induced blueshift of the optical transitions in GaInAsN/GaAs quantum wells. Appl. Phys. Lett..

[16] Kudrawiec R (2004). Photoreflectance evidence of multiple band gaps in dilute GaInNAs layers lattice-matched to GaAs. J. Appl. Phys..

[17] Kudrawiec R (2004). The energy-fine structure of GaInNAs/GaAs multiple quantum wells grown at different temperatures and postgrown annealed. J. Appl. Phys..

[18] Jin, Y. *et al* Influence of Si-N complexes on the electronic properties of GaAsN alloys. *Appl*. *Phys*. *Lett*. **95**, 092109-1-3 (2009).

[19] Lay TS (2004). Probing the electronic structures of III-V-nitride semiconductors by x-ray photoelectron spectroscopy. J. Vac. Sci. Technol. B.

[20] Liu HF (2004). *In situ* annealing effect on the structural properties of near-surface GaInNAs/GaAs quantum wells. J. Cryst. Growth.

[21] Kobayashi K (2009). Hard X-ray photoemission spectroscopy. Nuclear Instruments and Methods in Physics Research Section A: Accelerators, Spectrometers, Detectors and Associated Equipment. Nucl. Instr. Methods Phys. Res. A.

[22] Yumoto H (2009). Design optimization of highly accurate elliptical mirrors for hard-x-ray micro focusing probes at SPring-8. Proc. SPIE.

[23] Takamatsu D (2011). Nanoscale observation of the electronic and local structures of LiCoO2 thin film electrode by depth-resolved X-ray absorption spectroscopy. J. Phys. Chem. Lett..

[24] Taylor JA (1982). An XPS study of the oxidation of AlAs thin films grown by MBE. J. Vac. Sci. Technol..

[25] Hashizume T, Ikeya K, Mutoh M, Hasegawa H (1998). Surface passivation of GaAs with ultrathin Si_3_N_4_/Si interface control layer formed by MBE and *in situ* ECR plasma nitridation. Appl. Surf. Sci..

[26] Friedel P, Landesman JP, Boher P, Schneider J (1987). Cleaning and nitridation of GaAs surfaces in multipolar plasmas investigated by *insitu* photoemission and spectroscopic ellipsometry. J. Vac. Sci. Technol. B.

[27] Schön G (1973). Auger and direct electron spectra in X-ray photoelectron studies of zinc, zinc oxide, gallium and gallium oxide. J. Electron Spectrosc. Relat. Phenom..

[28] Contour JP, Massies J, Saletes A (1985). X-ray photoelectron spectroscopy study of GaAs (001) and InP (001) cleaning procedures prior to molecular beam epitaxy. Jpn. J. Appl. Phys..

[29] Sahra Gard F (2011). Construction and testing of a hydrogen cracking cell. Iran. J. Phys. Res..

[30] Ishikawa, F. *et al*. Direct observation of N-(group V) bonding defects in dilute nitride semiconductors using hard x-ray photoelectron spectroscopy. *Appl*. *Phys*. *Lett*., **98**, 121915-1-3 (2011).

[31] Procop M (1992). XPS data for sputter-cleaned In_0.53_Ga_0.47_As, GaAs, and InAs surfaces. J. Electron Spectrosc. Relat. Phenom..

[32] Zhang SB, Wei S-H (2001). Nitrogen solubility and induced defect complexes in epitaxial GaAs: N. Physical Review Letters.

[33] Laaksonen, K., Komsa, H. P., Rantala, T. T. & Nieminen, R. M. Nitrogen interstitial defects in GaAs. *J*. *Phys*.: *Condens*. *Matter* **20**, 235231-1-4 (2008). 10.1088/0953-8984/20/23/235231 21694322

[34] Fan WJ (2002). Comparison of nitrogen compositions in the as-grown GaN_*x*_As_1−*x*_ on GaAs measured by high-resolution x-ray diffraction and secondary-ion mass spectroscopy. Appl. Phys. Lett..

[35] Li W, Pessa M, Likonen J (2001). Lattice parameter in GaNAs epilayers on GaAs: Deviation from Vegard’s law. Appl. Phys. Lett..

[36] Reason M (2004). Mechanisms of nitrogen incorporation in GaAsN alloys. Appl. Phys. Lett..

[37] Ahlgren T, Vainonen-Ahlgren E, Likonen J, Li W, Pessa M (2002). Concentration of interstitial and substitutional nitrogen in GaN_*x*_As_1−*x*_. Appl. Phys. Lett..

[38] Chauveau JM, Trampert A, Ploog KH, Tournié (2004). Nanoscale analysis of the In and N spatial redistributions upon annealing of GaInNAs quantum wells. Appl. Phys. Lett..

[39] Corneille JS, He JW, Goodman DW (1994). XPS characterization of ultra-thin MgO films on a Mo (100) surface. Surf. Sci..

[40] Morifuji M, Ishikawa F (2016). Perturbation analysis on large band gap bowing of dilute nitride semiconductors. Physica B.

[41] Ishikawa, F., Höricke, M., Jahn, U., Trampert, A. & Ploog, K. H. Molecular beam epitaxial growth window for high-quality (Ga,In) (N,As) quantum wells for long wavelength emission. *Appl*. *Phys*. *Lett*. **88**, 191115-1-3 (2006).

[42] Däweritz, L. & Hey, R. *Surf. Sci*. **236**, 15–22 (1990).

[43] Kudrawiec, R. *et al* Temperature dependence of the optical transitions in Ga_0.64_In_0.36_N_0.046_As_0.954_ multiquantum wells of various widths studied by photoreflectance. *J*. *Appl*. *Phys*. **106**, 033507-1-5 (2009).

[44] Shinoda K (2009). Nondestructive depth resolved analysis by using grazing exit fluorescence-yield X-ray absorption spectroscopy. J. Surf. Anal..

[45] Higashi, K., Ishikawa, F., Handa, K., Emura, S. & Kondow, M. Epitaxial lift-off for sample preparation of x-ray absorption fine structure. *Rev*. *Sci*. *Instrum*. **81**, 043903-1-4 (2010). 10.1063/1.3355038 20441346

[46] Taguchi T, Ozawa T, Yashiro H (2005). REX2000: yet another XAFS analysis package. Physica Scripta..

[47] Sun YM, Sloan DW, McEllistrem M, Schwaner AL, White JM (1995). Surface chemistry of 1, 1]dimethylhydrazine on GaAs (100). J. Vac. Sci. Technol A.

[48] Wu TH, Su YK, Chuang RW, Cheng CY, Lin YC (2012). Characterization of the post-thermal annealing effect for p-GaAs/i-InGaAsN/n-GaAs hetero-junction solar cells. Solar Ener. Mater. Solar Cells.

[49] Veal TD, Mahboob I, Piper LFJ, McConville CF, Hopkinson M (2004). Core-level photoemission spectroscopy of nitrogen bonding in GaN_*x*_As_1−*x*_ alloys. Appl. Phys. Lett..

[50] Carin R, Deville JP, Werckmann J (1990). An XPS study of GaN thin films on GaAs. Surf. Interface Anal..

[51] Iwakuro H, Tatsuyama C, Ichimura S (1982). XPS and AES studies on the oxidation of layered semiconductor GaSe. Jpn. J. Appl. Phys..

[52] Faur M, Faur M, Jayne DT, Goradia M, Goradia C (1990). XPS investigation of anodic oxides grown on p]type InP. Surf. Interface Anal..

[53] King DE, Fernandez JE, Swartz WE (1990). An XPS study of the doping of trans, trans-p-distyrylbenzene with AsF5: a model conducting polymer system. Appl. Surf. Sci..

[54] http://xpssimplified.com/elements/gallium.php.

[55] Breeze PA, Hartnagel HL, Sherwood PMA (1980). An Investigation of Anodically Grown Films on GaAs Using X]Ray Photoelectron Spectroscopy. J. Electrochem. Soc..

