# Supplementary material for: Annealing induced atomic rearrangements on (Ga,In) (N,As) probed by hard X-ray photoelectron spectroscopy and X-ray absorption fine structure
Source: Sci Rep. 2018 Apr 13;8:5962. doi: 10.1038/s41598-018-23941-y (PMC5899128; doi:10.1038/s41598-018-23941-y)
Supplement: Supplementary file 1 — Supplementary information [file 41598_2018_23941_MOESM1_ESM.pdf]

Supplemental Information for:

Annealing induced atomic rearrangements on (Ga,In)(N,As) probed by hard X-ray photoelectron spectroscopy and X-ray absorption fine structure

Fumitaro Ishikawa[1,2], Kotaro Higashi[1], Satoshi Fuyuno[1],
Masato Morifuji[1], Masahiko Kondow[1], and Achim Trampert[3]
[1]Graduate School of Engineering, Osaka University, 2-1 Yamadaoka, Suita, Osaka 565-0871, Japan
[2]Graduate School of Science and Engineering, Ehime University,3 Bunkyo-cho, Matsuyama, Ehime 790-8577, Japan
[3]Paul-Drude-Institute für Festköperelektronik,Hausvogteiplatz 5-7, 10117 Berlin, Germany

Depth resolved XAFS measurements

Fig. S1 shows schematic illustrations of (a) the XAFS apparatus configurations and (b) the system used to perform depth-resolved measurements. The fluorescent X-rays are detected using a two-dimensional pixel array composed of a screen-type detector (Pilatus). Two-dimensional mapping of the fluorescent X-ray signals was also performed. By extracting signals from around the onset of the absorption edge intensity, we can detect the information from the material close to the sample surface alone.

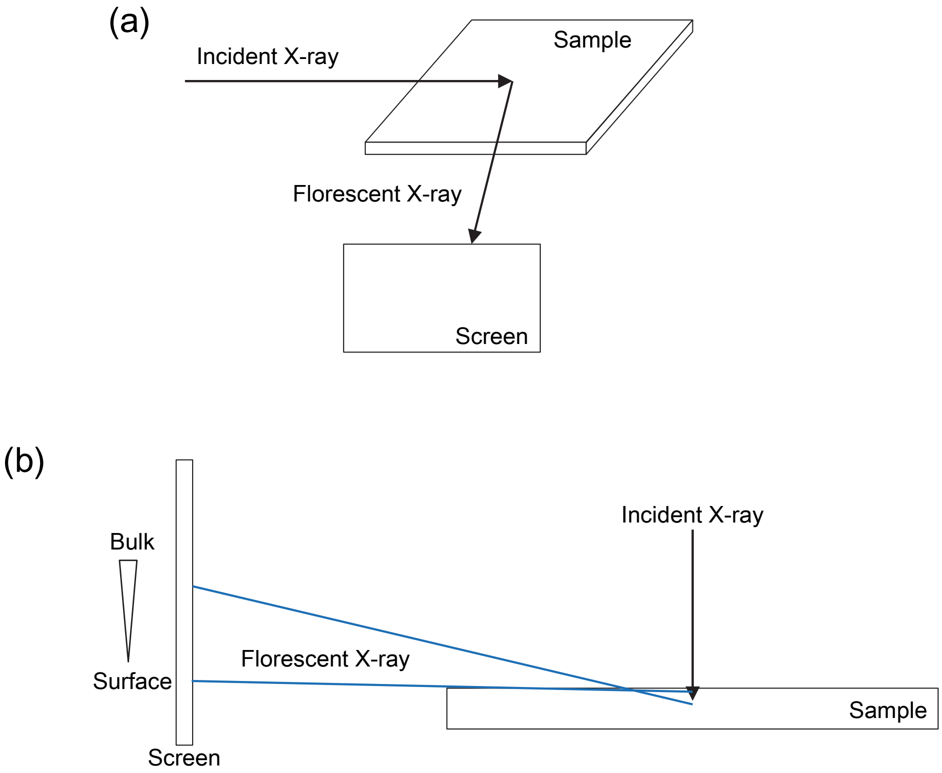

Fig. S1. Schematic illustrations of (a) the XAFS apparatus configurations, and (b) the system used to perform depth-resolved measurements.

XAFS measurements of Ga and In absorption edges

Figure S2 depicts the XAFS measurement conditions used for (a) Ga K-edges and (b) In K-edges. To measure the Ga-K edge, we used depth-resolved XAFS measurements unless the signal contained components from the bottom side GaAs layer, including the thick substrate, which would eclipse the information from the target (Ga,In)(N,As) layer. To measure the In K-edge, we performed standard fluorescence mode XAFS measurements. Because In is only included within the (Ga,In)(N,As) layer, this allows us to obtain the genuine information from the (Ga,In)(N,As) layer.

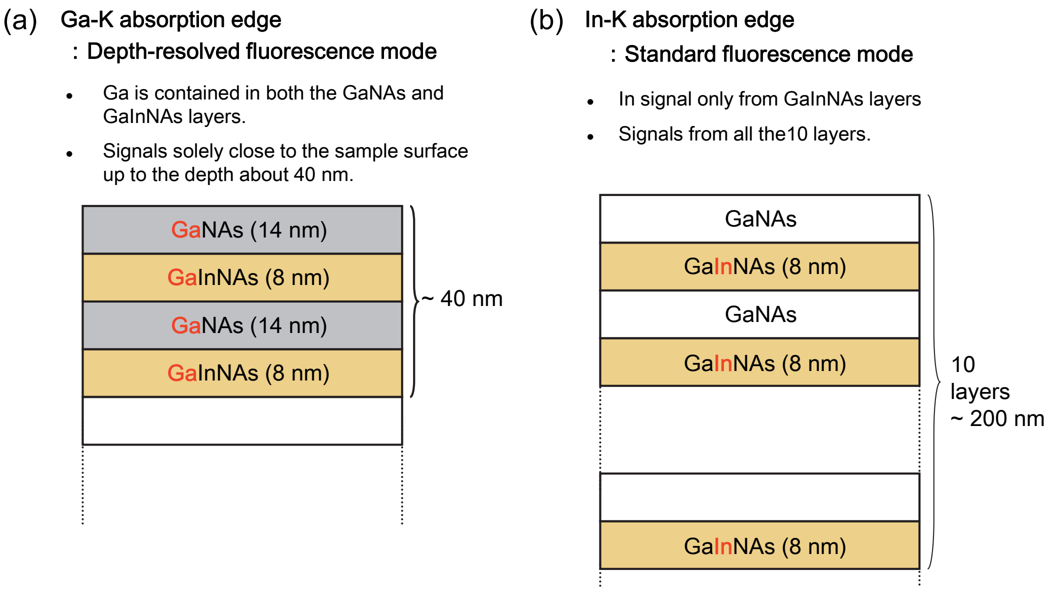

Fig. S2. Schematic illustration of XAFS measurement conditions for (a) Ga K-edges and (b) In K-edges.

Results of XAFS measurements

Figures S3 to S7 summarize the experimental and calculated polarization-dependent XAFS results, including the $\chi(k)k^3$ oscillation results and the RSFs for the Ga and In K-edges. The fitted parameters are summarized in Tables SI and SII for the series of experiments for the Ga and In K-edges, respectively.

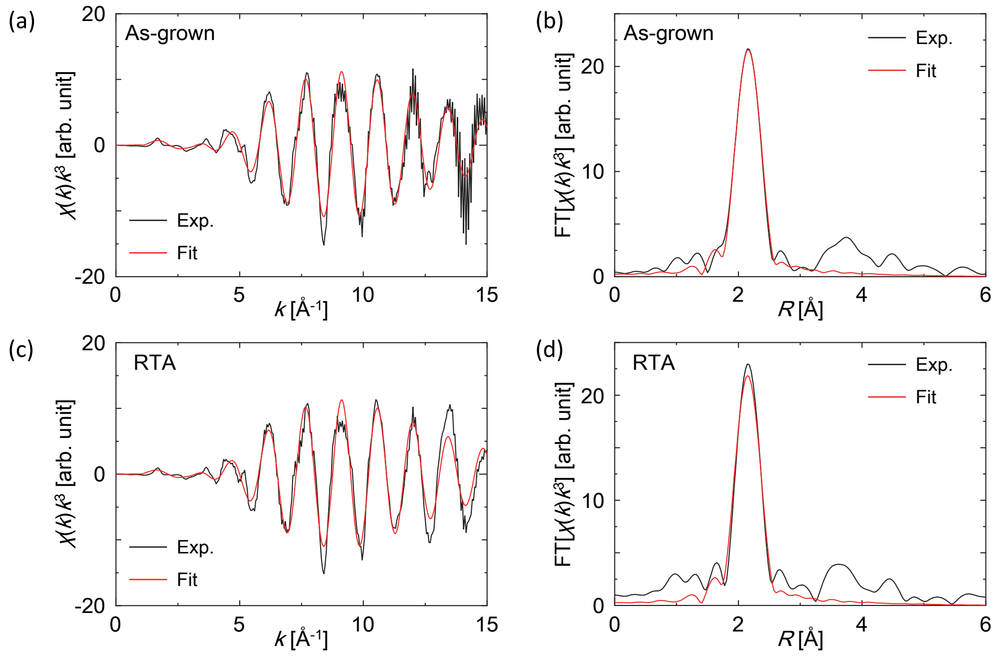

Fig. S3. Experimental (black) and calculated (red) characteristics for (a) $\chi(k)k^3$ oscillations and (b) the RSFs for the Ga K-edges for incident X-rays polarized in the [001] direction that were obtained from the as-grown sample. (c) and (d) show the corresponding quantities for the sample after RTA.

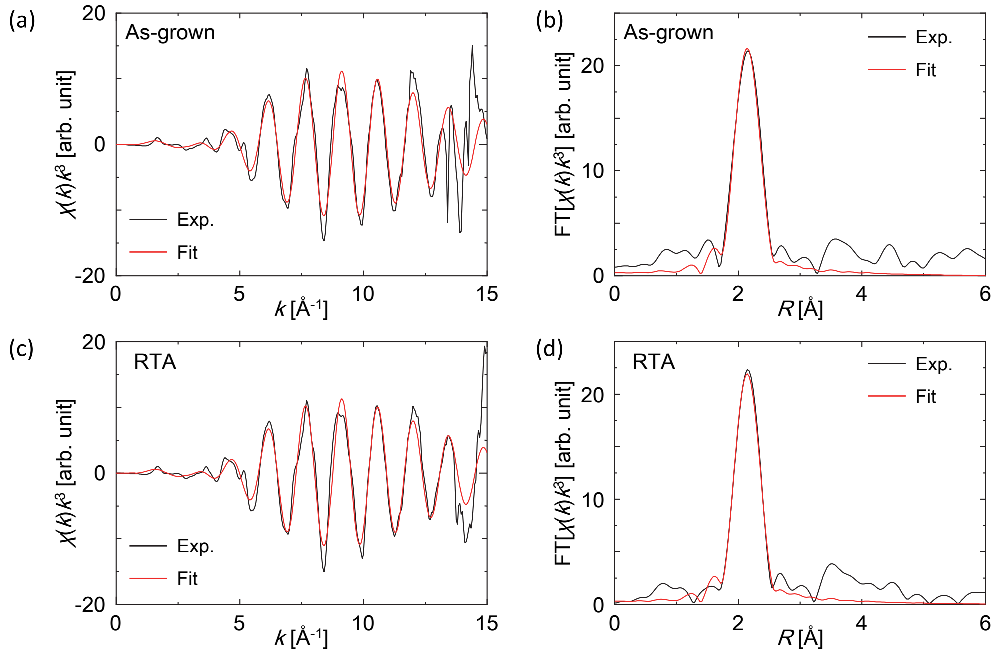

Fig. S4. Experimental (black) and calculated (red) characteristics for (a) $\chi(k)k^3$ oscillations and (b) the RSFs for the Ga K-edges for incident X-rays polarized in the [$\bar{1}$10] direction that were obtained from the as-grown sample. (c) and (d) show the corresponding quantities for the sample after RTA.

Table SI. Fitted parameters obtained from XAFS results on the Ga K-edges. The Debye-Waller factor value was fixed for the fitting procedure.

Sample		CN(Ga-As)	σ^2 [10^{-3} Å^2]	ΔE_0 [eV]	*R*(Ga-As) [Å]	R-factor [%]
// (001)	As-grown	3.89 ± 0.26	4.6	4.3 ± 1.9	2.45 ± 0.01	1.3
	RTA	3.94 ± 0.25	4.6	3.2 ± 1.9	2.45 ± 0.01	3.3
// ($\bar{1}$10)	As-grown	3.91 ± 0.49	4.6	2.2 ± 3.8	2.45 ± 0.01	3.3
	RTA	3.95 ± 0.25	4.6	2.3 ± 2.3	2.45 ± 0.01	1.4

k-range = 3 - 14 Å^{-1}, k-weight = 3, R-range = 1.0 – 3.0 Å,
CN: Coordination number, σ^2: Debye-Waller factor, ΔE_0: Energy sift, R: Bond distance

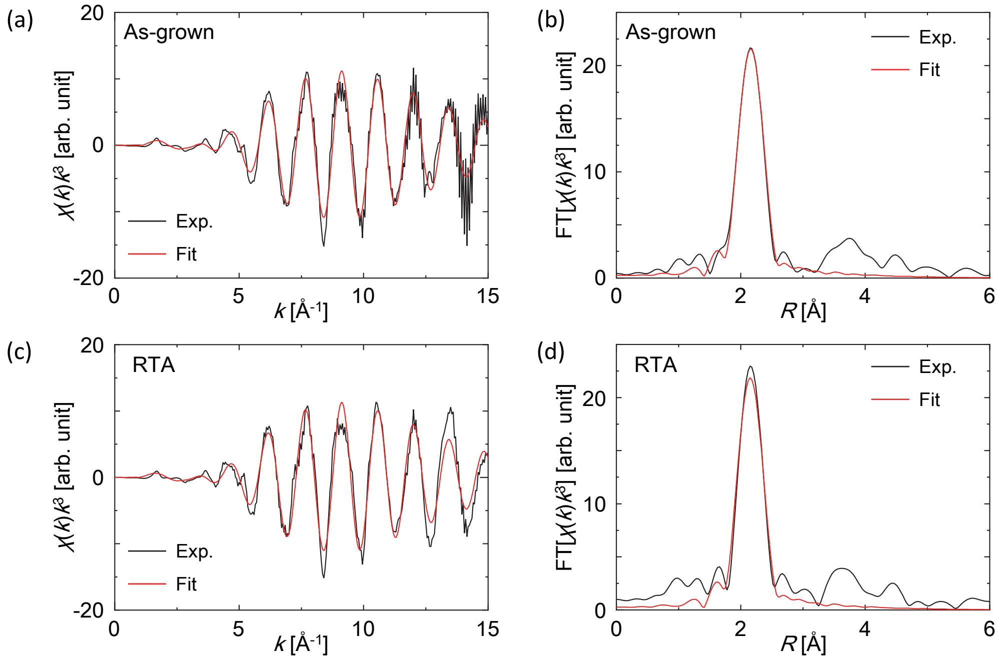

Fig. S6. Experimental (black) and calculated (red) characteristics for (a) $\chi(k)k^3$ oscillations and (b) the RSFs for the In K-edges for incident X-rays polarized in the [001] direction that were obtained from the as-grown sample. (c) and (d) show the corresponding quantities for the sample after RTA.

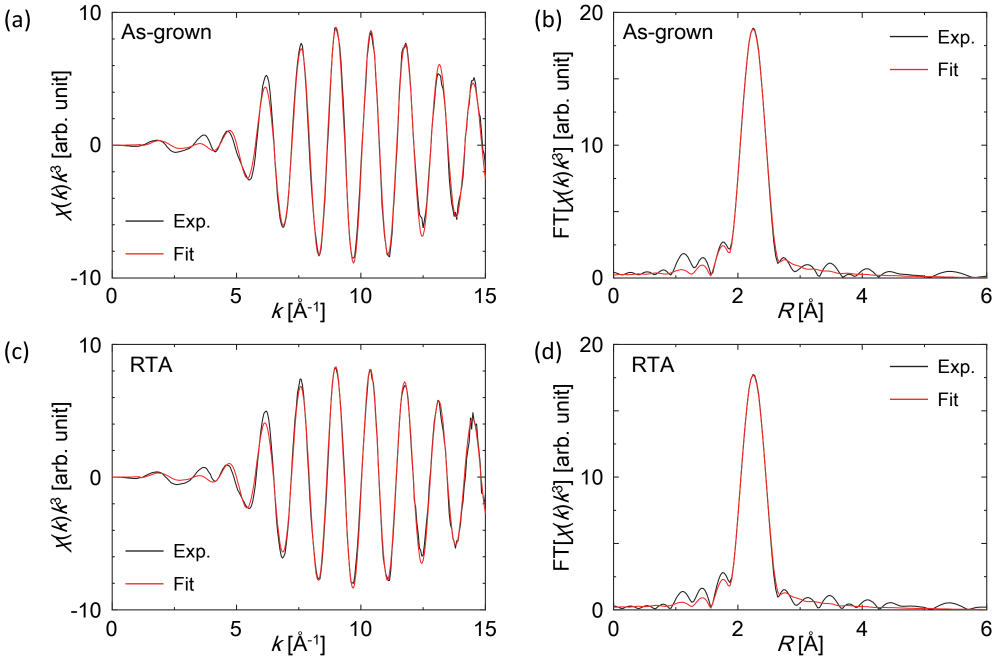

Fig. S7. Experimental (black) and calculated (red) characteristics for (a) $\chi(k)k^3$ oscillations and (b) the RSFs for In K-edges for incident X-rays polarized in the [110] direction that were obtained from the as-grown sample. (c) and (d) show the corresponding quantities for the sample after RTA.

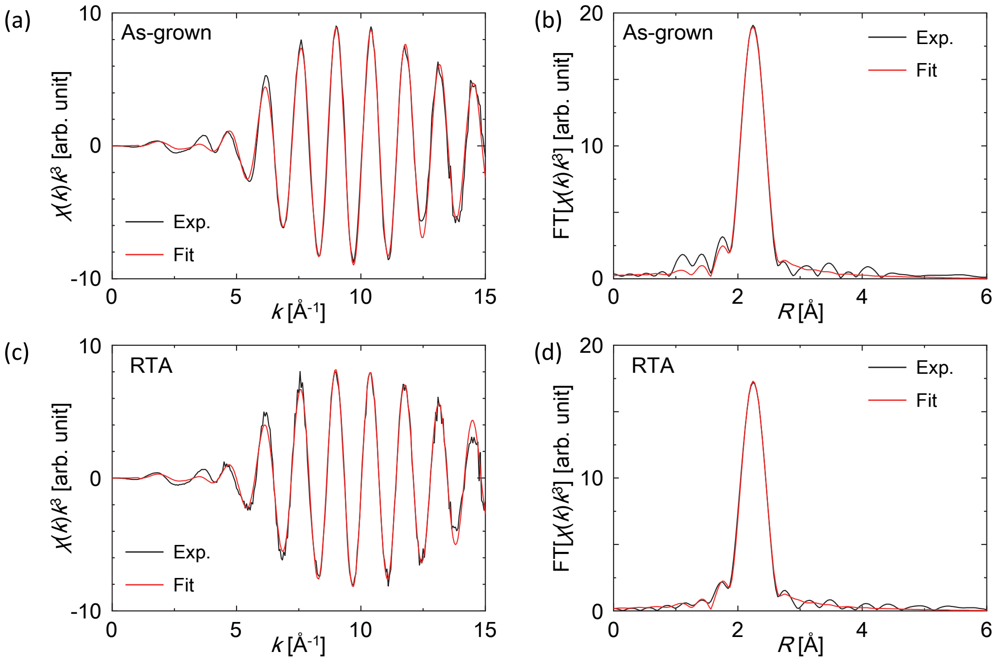

Fig. S8. Experimental (black) and calculated (red) characteristics for (a) $\chi(k)k^3$ oscillations and (b) the RSFs for In K-edges for incident X-rays polarized in the [$\bar{1}$10] direction that were obtained from the as-grown sample. (c) and (d) show the corresponding quantities for the sample after RTA.

Table SII. Fitted parameters obtained from the XAFS results on the In K-edges. The Debye-Waller factor value was fixed for the fitting procedure.

Sample		CN(In-As)	σ^2 [10^{-3} Å^2]	ΔE_0 [eV]	*R*(In-As) [Å]	R-factor [%]
// (001)	As-grown	3.84 ± 0.15	3.7	4.1 ± 1.5	2.58 ± 0.01	0.7
	RTA	3.64 ± 0.38	3.7	1.0 ± 4.2	2.57 ± 0.01	3.0
// (110)	As-grown	3.77 ± 0.13	3.7	5.1 ± 1.3	2.58 ± 0.00	0.6
	RTA	3.57 ± 0.11	3.7	4.8 ± 1.2	2.58 ± 0.00	0.6
// $(\bar{1}10)$	As-grown	3.80 ± 0.16	3.7	4.6 ± 1.5	2.58 ± 0.01	0.8
	RTA	3.49 ± 0.12	3.7	4.5 ± 1.2	2.58 ± 0.00	0.7

k-range = 3 - 15 Å^{-1}, k-weight = 3, R-range = 1.0 – 3.0 Å,
CN: Coordination number, σ^2: Debye-Waller factor, ΔE_0: Energy sift, R: Bond distance
